# Development and characterization of novel chimeric monoclonal antibodies for broad spectrum neutralization of rabies virus

**DOI:** 10.1371/journal.pone.0186380

**Published:** 2017-10-18

**Authors:** Pan Kyeom Kim, Sun Ju Keum, Modupe O. V. Osinubi, Richard Franka, Ji Young Shin, Sang Tae Park, Man Su Kim, Mi Jung Park, Soo Young Lee, William Carson, Lauren Greenberg, Pengcheng Yu, Xiaoyan Tao, Wang Lihua, Qing Tang, Guodong Liang, Madhusdana Shampur, Charles E. Rupprecht, Shin Jae Chang

**Affiliations:** 1 Celltrion, INC, Department of Research and Development, Incheon, Republic of Korea; 2 Centers for Disease Control and Prevention, Atlanta, Georgia, United States of America; 3 Chinese Center for Disease Control and Prevention, Beijing, China; 4 National Institute of Mental Health and Neurosciences, Bangalore, India; 5 LYSSA LLC, Cummings, Georgia, United States of America; Icahn School of Medicine at Mount Sinai, UNITED STATES

## Abstract

Current post-exposure prophylaxis for rabies virus infection has several limitations in terms of supply, cost, safety, and efficacy. Attempts to replace human or equine rabies immune globulins (HRIG or ERIG) have been made by several companies and institutes. We developed potent monoclonal antibodies to neutralize a broad spectrum of rabies viruses by screening hybridomas received from the U.S. Centers for Disease Control and Prevention (CDC). Two kinds of chimeric human antibodies (chimeric #7 and #17) were constructed by cloning the variable regions from selected hybridomas and the constant region of a human antibody. Two antibodies were bound to antigenic site III and I/IV, respectively, and were able to neutralize 51 field isolates of rabies virus that were isolated at different times and places such as Asia, Africa, North America, South America, and Australia. These two antibodies neutralize rabies viruses with high efficacy in an *in vivo* test using Syrian hamster and mouse models and show low risk for adverse immunogenicity.

## Introduction

Rabies is an acute viral encephalomyelitis caused by lyssaviruses. The prototypical species, rabies virus (RABV), is a member of the genus *Lyssavirus* in the family *Rhabdoviridae*. *Lyssavirus* is one of seven genera in this family, comprising classical RABV (genotype 1), Lagos bat virus (LBV; genotype 2), Mokola virus (MOKV; genotype 3), Duvenhage virus (DUVV; genotype 4), European bat lyssavirus 1 (EBLV-1; genotype 5), European bat lyssavirus 2 (EBLV-2; genotype 6), and Australian bat lyssavirus (ABLV; genotype 7). Recently, four additional viruses, isolated from insectivorous bats, have been proposed as new members of the *Lyssavirus* genus: Aravan virus (ARAV), Khujand virus (KHUV), Irkut virus (IRKV), and West Caucasian bat virus (WCBV) and other putative members have been characterized: Bokeloh bat lyssavirus(BBLV), Gannoruwa bat lyssavirus(GBLV), Shimoni bat virus(SHIBV), Lleida bat lyssavirus(LLEBV), and Ikoma lyssavirus(IKOV) [1–6]. The most common route of RABV transmission is through the bite of infected mammals. Occurrence of rabies after bites by rabid canids ranges from 40% to 80% and depends in part on the severity and location of the wound, as well as the concentration of virus in the saliva [7, 8]. However, after development of illness, almost all humans infected by RABV succumb (fatality rate > 99%) [9]. Globally, rabies causes about 60,000 human deaths every year, with most cases occurring in Asian and African countries [10–12].

Treatment after category III exposure, single or multiple transdermal bites or scratches, is immediate administration of rabies post-exposure prophylaxis (PEP) comprising a RABV vaccine to induce virus neutralizing antibodies and RABV immune globulin (RIG) for passive protection [5,13,14]. Currently available RIGs for clinical use originate from horses (ERIG) or humans (HRIG). These RIGs are produced from pooled sera of donors immunized with rabies vaccines [15, 16]. Although ERIG is more affordable than HRIG, several adverse reactions have been reported [17, 18]. Therefore, HRIG is recommended for PEP in developed countries. However, the cost of HRIG is approximately five times higher than that of ERIG, and the supply is limited. In addition to problems of cost and supply, there is the risk a potential of contamination from blood products from humans or other animals, including unknown agents and pathogens [19]. To overcome these issues, manufacturers and research groups have tried to produce alternatives to current RIG. Several human and murine monoclonal antibodies (MAbs) are being investigated. These MAbs target the RABV glycoprotein (G) protein, neutralize virus *in vitro* and protect animals against lethal RABV challenge [20–24].

The RABV G protein is a single transmembrane protein that is assembled as a trimeric spike. When RABV infects susceptible cells, this G protein initially binds to host cell surface receptors [1]. In previous studies, the antigenic sites of RABV G protein were characterized using MAbs and their respective neutralization-resistant virus variants [25–29]. Antigenic site I is located at amino acid position 226–231 and harbors both conformational and linear epitopes [30]. Antigenic site II is a discontinuous conformational epitope at residue 34–42 (IIb) and 198–200 (IIa). Site III is located at position 330–338, and site IV is one amino acid at position 251. Minor site “a” is located at position 342–343. The V antigenic site is a linear epitope at residues 261–264, which includes antigenic site VI, which is a one amino acid at position 264 [25–31].

Recently, several murine and human MAbs were produced by different methods, including the human-mouse hybridoma method and phage display technology. These MAbs targeted distinct antigenic sites. There were two MAbs that bound to antigenic site I, referred to as CR57 [22] and 62-71-3 [32]. CR57 is a human MAb and recognized linear epitope within antigenic site I (amino acids 226 to 231), with critical binding residues at positions 226, 228, 229, and 230 [22]. Chimeric MAb 62-71-3 was produced originally in mice and later in the plant *Nicotiana benthamiana*. A critical role in virus neutralization was identified for antigenic site I of the RABV G protein, as well as two amino acids (K226 and G229) within site I, of MAb 62-71-3 [32]. Four mouse MAbs, E559.9.14, 1112–1, M727-5-1, and M777-16-3, identified by a group of World Health Organization (WHO) collaborating centers, recognized epitopes in antigenic site II [13]. Human MAbs CR4098, RAB1, and 17C7, developed by individual research groups, targeted antigenic site III [23,33,34]. Monoclonal antibody 110–3 described at Dietzschold et al. targeted antigenic site IV [35]. Finally, a MAb cocktail CL-184 comprising of two human MAbs, CR57 and CR4098, has been developed and well characterized *in vitro* and *in vivo*, and has shown safety and tolerability in phase I/II clinical trials [21,24,36].

In the present study, to develop potent MAbs against a broad spectrum of RABV, the neutralizing MAb activities from several hybridomas of the U.S. CDC were evaluated, and two potent hybridomas which produced MAbs neutralized various field isolates of RABV were selected. The IgG variable regions from selected hybridomas were amplified and cloned into vectors containing a human antibody constant region. Stable cell lines producing two chimeric MAbs were established. We confirmed the binding affinities of the purified MAbs to RABV G protein and their neutralizing activities against 51 variants of RABV, including isolates from India and China. The antigenic sites of established MAbs were identified by surface plasmon resonance (SPR) and *in vitro* escape viruses. To confirm efficacy, we conducted *in vivo* tests in hamster and mouse challenge models. The Immunogenicity of these MAbs was determined by measuring CD4+ T cell responses.

## Materials and methods

### Hybridoma cells

Based on previous research and the availability of hybridomas at the U.S. CDC, the neutralizing activities of 13 MAbs were tested and those showing promising neutralizing activities were selected.

### Standard rapid fluorescent focus inhibition test (RFFIT)

The RFFIT was performed as described [37]. Briefly, five-fold serial dilutions of MAbs were incubated with the RABV CVS-11 strain in 8-well tissue culture chamber slides for 90 min at 37°C. Mouse neuroblastoma (MNA) cells were added to the sample-virus mixture and incubated for an additional 20 to 24 h at 37°C with 2 to 5% CO_2_. Slides were fixed in acetone and stained with an anti-rabies N-FITC conjugate. Twenty distinct microscopic fields per well were examined using a fluorescence microscope at a magnification of 160X to count the RABV-infected cells (foci). The number of positive fields with RABV-infected cells per well was recorded. The neutralization endpoint titer was defined as the highest sample dilution at which 50% of the observed microscopic fields contained one or more RABV-infected cells. The RFFIT titers were interpolated mathematically using the Reed and Muench method. The endpoint neutralization titer of the test serum was then transformed into international units (IU)/mL by calibrating results against the endpoint neutralization titers of the U.S. Standard Rabies Immune Globulin (SRIG) (lot R-3, 59 IU), which was determined in the same assay run, with an assigned potency value of 2.0 IU/ml.

### Fluorescent antibody virus neutralization (FAVAN) assay

The FAVN was performed as described [38]. Three-fold serial dilutions of MAb were incubated with 100 TCID_50_/well diluted CVS-11 in 96-well tissue culture plates for 1 h at 37°C and 5% CO_2_. BHK cells were added to the sample-virus mixture and incubated for 48–60 h at 37°C and 5% CO2. Plates were fixed by adding 80% acetone and stained with anti-rabies MAb (Median Diagnostics, Catalog No. 9061) using an ABC kit (Vector Laboratories, Catalog No. PK-4002) and DAB peroxidase (HRP) substrate kit (Vector Laboratories, Catalog No.SK-4100). A D_50_ endpoint titer (i.e., the point at which 50% of the wells at that serum dilution show the presence of virus) was calculated by the Spearman-Karber method. By convention, this titer is converted to a value in IU/mL by comparison to a standard reference serum with a known titer.

### Cloning of chimeric MAb #7 and #17

Total RNA from hybridoma cell line #2-21-23 and #62-71-3 was extracted using an RNeasy Plus Mini-Kit (Qiagen, 74134). Total cDNA synthesis with a specific sequence in the 5ʹ end was performed with a SMARTer RACE (Rapid Amplification of cDNA Ends) cDNA Amplification Kit (Clontech, 634925) using the isolated total RNA as a template. Finally, cDNA containing the whole variable region from hybridoma cell lines #2-21-23 and #62-71-3 was amplified using an Advantage2 PCR Kit (Clontech, 639207).

The cDNA fragments containing the entire variable regions of heavy and light chains were cloned into a TA vector using a TOPO TA Cloning Kit (Invitrogen, K4500), and then the DNA sequences of the variable regions of MAbs were analyzed by DNA sequencing.

Based on the DNA sequences of variable regions, chimeric MAb #7 and #17 were cloned into CMV promoter based expression vector(US 8772021) by connecting the variable region of the mouse hybridoma MAbs (#2-21-23 and #62-71-3) to the constant region of human IgG1 kappa by overlapping PCR, with confirmation by DNA sequence analysis.

### Expression of chimeric MAb #7 and #17

For transfection, CHO-K1 cells were seeded in 6-well plates and allowed to attach overnight. The DNA of the chimeric MAb #7 and #17 expression vectors was transfected into the cells using Lipofectamine LTX Reagent (Invitrogen, Cat. No. 15338), following manufacturer’s instructions.

The transfected cells were seeded onto 96-well plates using SFM4CHO media with 500nM methotrexate (MTX) 3 days after transfection. These cells were cultured in a humidified 5% CO_2_ incubator at 37°C. When the size of a cell patch reached over 70% of a well under MTX selection condition, the expression of chimeric MAb #7 and #17 in culture fluid were determined by enzyme-linked immunosorbent assay (ELISA). Chimeric MAb #7 and #17 expression cell clones that showed the highest expression of chimeric MAb #7 and #17 were selected by limited dilution method under 100nM MTX. The single cells selected were cultured in Celltrion proprietary media.

### Antibody titer analysis

A standard sandwich ELISA assay was used to assess the production levels of the secreted chimeric MAb #7 and #17. Microtiter plates (NUNCLON, Cat.# 49824) were coated with goat anti-human IgG γ-chain-specific (Jackson Immuno-Research, 109-006-098) antibody. Plates were blocked with 1% bovine serum albumin (BSA; Sigma, Cat.# A3803) in phosphate buffered saline (PBS; Sigma, Cat.# D5652). The captured product was detected using horse radish peroxidase (HRP)-conjugated goat anti-human IgG κ-chain (Sigma, Cat.# A7164) and substrate solution TMB (Sigma, Cat# T0440), and 1 N sulfuric acid was used to stop the reaction. The absorbance was measured at OD 450/650 nm using an ELISA plate reader (SPECTRAmax plus384, Molecular Devices). The titer of MAb #7 and #17 was calculated by comparison to a standard curve using a purified human IgG standard (IgG kappa specific, Sigma, I5154).

### RABV G protein purification

After 4 days of RABV ERA strain infection of BHK cells, supernatant of virus-infected cells was harvested, and virus particles were collected by ultracentrifugation for 120 min at 50,000 × *g* and 4°C. The virus particle pellet was resuspended in NTE buffer at pH 7.5. Virus was isolated by 15–50% sucrose density gradient for 1 h at 100,000 × *g* and 4°C. After centrifugation, the virus particles were obtained at a buoyant density of 1.17 g/cm^3^, and 0.3 mol/L NaCl, 50 mmol/L trometamol-HCl (pH 7.6), and 2% OGP were added to the purified RABV. The mixture was incubated for 20 min at room temperature and then centrifuged for 70 min at 120,000 × *g*. The solubilized G proteins were purified by isopycnic centrifugation on a sucrose gradient for 36 hrs at 150,000 × *g* and 4°C. After centrifugation, the RABV G protein was collected from the bottom of the tubes using a hypodermic needle. The concentration of purified RABV G protein was measured using micro BCA protein assay kit (Thermo, Prod #23235).

### RABV G protein ELISA

Purified RABV G protein (1 μg/mL) was coated on a plate overnight at 4°C, and non-specific binding was reduced by inoculation of a diluent buffer for 1 hr at room temperature. Ten-fold serially diluted chimeras #7, #17, and HRIG were added to the G protein-coated plate for 1 h at room temperature and then stained with anti-human kappa light chain-peroxidase (Sigma, Catalog No. A7164).

### Epitope mapping

An SPR analysis for epitope mapping was performed at 25°C using a CM5 sensor chip in a BIAcore T200 system. HBS-EP buffer (GE Healthcare) was used as the running buffer during analysis. Purified RABV G protein was immobilized on the sensor chip using the suggested amine coupling procedure. Each antibody (1 μM) was injected and bound to the immobilized RABV G protein via a given association phase (120-s) at a constant flow rate of 30 μL/min. After a 100-s period of buffer flow, the secondary antibody (1 μM) was injected, followed by elimination of accumulated antibodies with the optimal regeneration buffers 10 mM glycine-HCl (pH 2.0) and 1 mM sodium hydroxide. At the end of each antibody association phase, the changed RU levels compared to each baseline were determined as a measure for binding.

### Generation of escape viruses and cDNA sequencing

An experiment to generate MAb escape viruses was performed according to a previously described method [22]. Serial dilutions of CVS-11 ranging from 10^−1^ to 10^−7^ TCID50/mL were incubated with an appropriate amount (1 IU/mL) of MAb chimeric #7 or #17 for 1 h at 37°C and 5% CO_2_. The mixtures were added to BHK-21 cells, and then potential escape viruses were harvested after 3 days of incubation in the presence of 1 IU/mL of MAbs chimeric #7 or #17. The cells on the plates were fixed and incubated with antibodies against the RABV nucleoprotein, followed by staining with FITC-conjugated goat anti-mouse immune globulin G (IgG). Supernatants from wells showing one to four fluorescent foci were used to infect BHK-21 cells for virus amplification because the escape virus were well isolated in virus harvested from small number of foci (one to four) than to that harvested from more foci, empirically. The survival of the amplified virus was verified in the presence of 4 IU/mL MAbs. The identified escape mutants were amplified, and total RNA was isolated using an RNeasy Mini Kit (Qiagen) according to the manufacturer’s instruction. Subsequently, reverse transcription-PCR (RT-PCR) was performed using RABV-specific primers and a One-step SuperScript RT-PCR Kit with Taq DNA polymerase (Takara). cDNA was then sequenced by standard procedures.

### *In vivo* hamster challenge model

Two-month-old Syrian hamsters (weight 80–100g, Charles River Laboratories) were infected with mouse intracerebral lethal dose 50 (MICLD_50_) of RABV isolated from a dog in China (RV342, 10^5^TCID_50_/ml) on day −1. Chimeric MAbs #7, and #17 or HRIG (Imogam Rabies-HT, Sanofi Pasteur) at a dose 120 IU/kg (0.084mg/kg), 20 IU/kg (0.13mg/kg) or 12 IU/kg were administered on day 0. Hamsters were checked daily for clinical signs of rabies, which resulted in euthanasia by CO2 intoxication. Brains were removed at necropsy to detect RABV antigens by the direct fluorescent antibody test. Animals were followed to day 45 post infection (pi). In a study of the chimeric MAb administration with vaccine (Human diploid Imovax, Sanofi Pasteur), the RABV vaccines were administered on days 0, 3, 7, and 14 after infection. This Syrian hamster challenge study was performed by the U.S. CDC. Animal care and experimental procedures were approved by the CDC Institutional Animal Care and Use Committee (IACUC) and were performed in compliance with the CDC Institutional Animal Care and Use Guidelines. The IACUC protocol number for the project was 2123FRAHAMC.

### *In vivo* mouse challenge study

The mouse challenge study of RABV isolated from India was performed by the National Institute of Mental Health and Neurosciences (NIMHANS) in Bangalore, India. The field isolates were obtained from the brains of different animals from 2010 to 2011 by mouse inoculation as recommended by the WHO (SOP NIMH/NV/RAB 006). The RABV had been stored as mouse brain homogenate (20%) and titrated by inoculation of virus in young Swiss Albino mice (10 mgs). A virus dose of 100 LD_50_ was used to challenge mice and chimera #7 or #17, at a dose of 20 IU/kg and 40 IU/kg, respectively, were injected into the same area where RABV was inoculated (8 mice/group). Mice were checked daily for clinical signs of rabies, which resulted in euthanasia using an overdose of anesthesia (Halothane). Animal care and experimental procedures were approved by the NIMHANS Institutional Animal Ethics Committee (IAEC). The IAEC number of this project was IAEC/44/271/N.V.

### Immunogenicity test

The immunogenicity of MAb chimeras #7 and #17 was determined by Antitope in Cambridge, UK. Peripheral blood mononuclear cells (PBMCs) were isolated from healthy community donor buffy coat (from blood drawn within 24 hours) obtained from UK National Blood Transfer Service (Addenbrooke’s Hospital, Cambridge, UK) and according to approval granted by Addenbrooke’s Hospital Local Research Ethics Committee. A cohort of 50 donors was selected to best represent the number and frequency of HLA-DR allotypes expressed in the world population. PMBSs obtained from selected 50 donors were plated on 24-well plates individually, and chimeras #7 and #17 were added to PBMCs for a final concentration of 50 μg/mL. Cultures were incubated for a total of 8 days at 37°C with 5% CO_2_. On 5, 6, 7, and 8 days, the cells were transferred to round-bottomed 96-well plate. The cultures were pulsed with 0.75 μCi [^3^H]-thymidine (Perkin Elmer, Beaconsfield, UK) and incubated for another 18 h before harvesting in filter mats using a TomTec Mach III cell harvester. Counts per minute (cpm) were determined by Meltilex (Perkin Elmer) scintillation counting on a 1450 Microbeta Wallac Trilux Liquid Scintillation Counter (Perkin Elmer) in paralux with low background counting. Keyhole Limpet Haemocyanin (KLH, Pierce, Cramlington, UK) was used as a positive control.

ELISpot plates (Millipore, Watford, UK) were pre-wetted and coated overnight with IL-2 capture antibody (R&D Systems, Abingdon, UK) in PBS. Non-specific binding was reduced by incubating with blocking buffer (1% BSA). PBMCs/well (4–6 × 10^5^) were plated, and chimeras #7 and #17 and a negative control (medium alone) and positive control (Phytohaemagglutanin (PHA), Sigma, Poole, UK) were added. After 8 days of incubation, biotinylated detection antibody, streptavidin-AP, and BCIP/NBT substrate were used to develop the ELISpot plates. Dried plates were scanned on an Immunoscan Analyser, and spots per well (spw) were determined using Immunoscan Version 4 software.

## Results

### Selection of hybridoma cells producing highly reactive anti-rabies MAbs

There were several hybridoma cell lines that were verified to produce MAbs with a neutralizing effect on a wide range of RABV by the U.S. CDC. To select the hybridoma cells that produced highly reactive MAbs against RABV, the neutralizing activities were evaluated by RFFIT on CVS-11 as described in the Materials and Methods. The media from each cell culture under the same conditions was used to measure neutralization of RABV. The neutralizing efficiency of each MAb produced by each hybridoma was expressed as IU per ml (Table 1). The top five clones, #2-21-8, #2-21-23, #2-21-14, #62-71-3, #62-80-6, displaying supernatants with high RFITT titers (IU/ml) were selected as candidate clones to produce potent anti-rabies MAbs. Clone #2-21-8 supernatant had the highest virus neutralizing titer (VNT), 150 IU/ml, and the other five selected clones had supernatants with VNTs higher than 0.7 IU/ml.

**Table 1:** Supernatant neutralizing activity of each hybridoma clone against CVS-11 virus.

ID	Isotype	Virus neutralizing titer (VNT, IU/mL)
2-21-8	IgG2a	150
2-21-23	IgG2a	83
2-21-14	IgG2a	17.8
62-71-3	Not tested	5.7
62-80-6	IgG2b	0.7
62-62-5	IgG2a	0.7
2-21-20	IgG2a	0.1
62-105-06	Not tested	0.03
62-111-1	IgG2a	0.03
62-114-6	IgG3	0.03
62-139-2	IgG1	0.03
2-21-17	Not tested	0.03
62-28-5	IgG2a	0.03

Neutralizing activity of supernatant produced by each hybridoma clone was determined in a standard RFFIT.

### Supernatant neutralizing ability of selected clones against various RABV

Generally, MAb utility is defined not only by the activity of neutralization, but also by its wide-spectrum reactivity to RABV. The U.S. CDC has more than 50 RABVs in storage from around the world. In an early stage of selecting the hybridomas, we analyzed the breadth of MAb neutralizing ability using a broad panel of street RABVs. Among the hybridomas, five candidates were identified, and then two hybridoma cell lines were selected based on their supernatant neutralization rates against test viruses (S1 Table). In the case of #2-21-14, the neutralizing activity was measured in only half of the viruses tested, and this hybridoma was excluded. The supernatant neutralization rates of the remaining four hybridoma cell lines were 76% (#2-21-8), 86% (#2-21-23), 92% (#62-71-3), and 57% (#62-80-6). Supernatant #2-21-23, #62-71-3 showed the highest neutralization rates.

### Generation and characterization of chimeric MAbs derived from hybridomas #2-21-23 and #62-71-3

To isolate potent anti-rabies MAbs from the selected hybridoma cells, we established a synthesized cDNA library of the variable region of antibodies produced by hybridoma cells #2-21-23 and #62-71-3. Chimeric MAbs were constructed by cloning the above established variable regions into a vector containing a constant region of human antibodies. The purified chimeric MAbs #2-21-23 and #62-71-3 were designated as chimeric MAb #7 and #17, respectively, and stable cell lines producing the two chimeric MAbs were established following the procedure described in the Material and Methods. The neutralizing efficiency of both purified chimeric MAbs was determined against the 45 viruses shown in S1 Table, an additional 6 viruses isolated from different hosts or times in southern India. This result showed that chimeric MAbs #7 and #17 demonstrated broad neutralization against the full RABV panel (50/51), and chimeric MAbs #7 and #17 complemented each other to neutralize RABVs that were neutralized weakly. The exception was one RABV isolate from a skunk in California, USA (Table 2).

**Table 2:** Neutralization breadth by chimeric MAbs #7 and 17 against RABV field isolates.

No	Lyssavirus (Isolated source, country)	Identification number* or Year, Date of isolation	Chimeric MAb #7	Chimeric MAb #17
1	Rabid Cow, France	1882^#^	≥^a^ 1120%	200%
2	Mongoose, South Africa	A07-0434	≥1555%	189%
3	Skunk, California, USA	A12-1826	<^b^ 6%	<6%
4	Dog, Tunissia	A07-0433	≥1120%	960%
5	Dog, Gabon, Africa	A04-2030	1040%	45%
6	Gray fox, Texas, USA	A04-0717	≥1120%	640%
7	Dog, Thailand	A04-2031	≥1120%	960%
8	Dog, Mexico	A04-2029	≥1120%	288%
9	Human/dog, Philippines	A07-0448	≥1120%	432%
10	Bat, Mexico	NA^@^	≥1120%	232%
11	Bat, Brazil	A04-2032	≥2240%	152%
12	Dog, Philippines	A07-0447	≥1120%	1040%
13	Bat, Washington, USA	A04-0723	≥1120%	880%
14	Bat, California, USA	A07-0449	17%	880%
15	Skunk, Texas, USA	A04-0714	≥1555%	18%
16	Raccoon, southeast, USA	A04-0712	≥1555%	233%
17	Dog, China	A07-0445	≥2240%	2080%
18	Cow/dog, China	A07-0446	≥2240%	≥2240%
19	Human/dog, United Kingdom	A07-0439	≥2240%	704%
20	Bat, Alabama, USA	A04-0720	≥1120%	256%
21	Bat, Pennsylvania, USA	A04-2024	≥1120%	40%
22	Bat, Alabama, USA	A04-2023	≥1120%	224%
23	Bat, Arizona, USA	A04-0721	23%	≥1120%
24	Bat, Virginia, USA	A07-0454	272%	1040%
25	Bat, Tennessee, USA	A07-0456	10%	500%
26	Bat, Tennessee, USA	A07-0450	640%	960%
27	Skunk, Texas, USA	A07-0457	≥1555%	62%
28	Arctic Fox, Alaska, USA	A04-0711	880%	68%
29	Raccoon dog, Russia, Far East	A07-0436	≥2240%	1600%
30	Dog, India	A07-0438	≥2240%	Not tested
31	Mongoose, Puerto-Rico	A04-2027	1040%	224%
32	Skunk, Wisconsin, USA	A04-0713	≥1555%	694%
33	Dog /Coyote, Texas, USA	A04-2022	≥1555%	Not tested
34	Human/wolf, Russia, Arctic	A07-0437	880%	880%
35	Bat, Tennessee, USA	A07-0455	880%	1120%
36	Dog, India	A07-0443	≥2240%	Not tested
37	Bat, Tennessee, USA	A07-0452	48%	≥1120%
38	Cow, Sri Lanka	A07-0440	≥2240%	1600%
39	Bat, Washington, USA	A04-0724	1040%	1040%
40	Bat, Australia	NA^@^	1040%	136%
41	Bat, Australia	NA^@^	≥1120%	880%
42	Dog, Argentina	1979^#^	≥1120%	880%
43	Coyote, Texas, USA	1991^#^	≥1555%	1333%
44	Bat, New York, USA	1982^#^	Not tested	≥1555%
45	Gray Fox, Arizona, USA	1986^#^	≥1120%	224%
46	Dog, Bangalore, India	2010.02.02^&^	≥100%^c^	≥100%^c^
47	Canine, Bangalore, India	2010.02.10^&^	≥100%^c^	≥100%^c^
48	Human, Koppal, India	2010.09.13^&^	≥100%^c^	≥100%^c^
49	Canine, Trivandrum, India	2011.07.18^&^	≥100%^c^	≥100%^c^
50	Canine, Trivandrum, India	2011.07.18^&^	≥100%^c^	≥100%^c^
51	Canine, Trivandrum, India	2011.10.14^&^	≥100%^c^	≥100%^c^

Neutralizing potency against each RABV field isolates was measured in a standard RFFIT and indicated as follows

% = (Neutralizing potency of chimeric MAb #7 or #17/ neutralizing potency of HRIG) x 100

^a^: The accurate neutralizing potency of chimeric Mab #7 or #17 could not be determined because the chimeric Mab #7 or #17 neutralized the viruses at the highest dilution factor in the tested range.

^b^: There was no neutralizing potency in the tested range.

^c^: The neutralizing potency was measured by relative comparison among HRIG, chimeric #7, and #17.

This means that chimeric Mab #7 or #17 have equal or higher neutralizing potency compared to HRIG.

*: Identification number used to record the year of the virus collection (e.g. A04-2027, collected in 2004 and the last four numbers are automatically generated by CDC accessioning system)

^#:^ The year of the virus collection

^&^: The date of the virus collection

^@^: There is no information for year/date of the virus isolation

**Fig 1:**
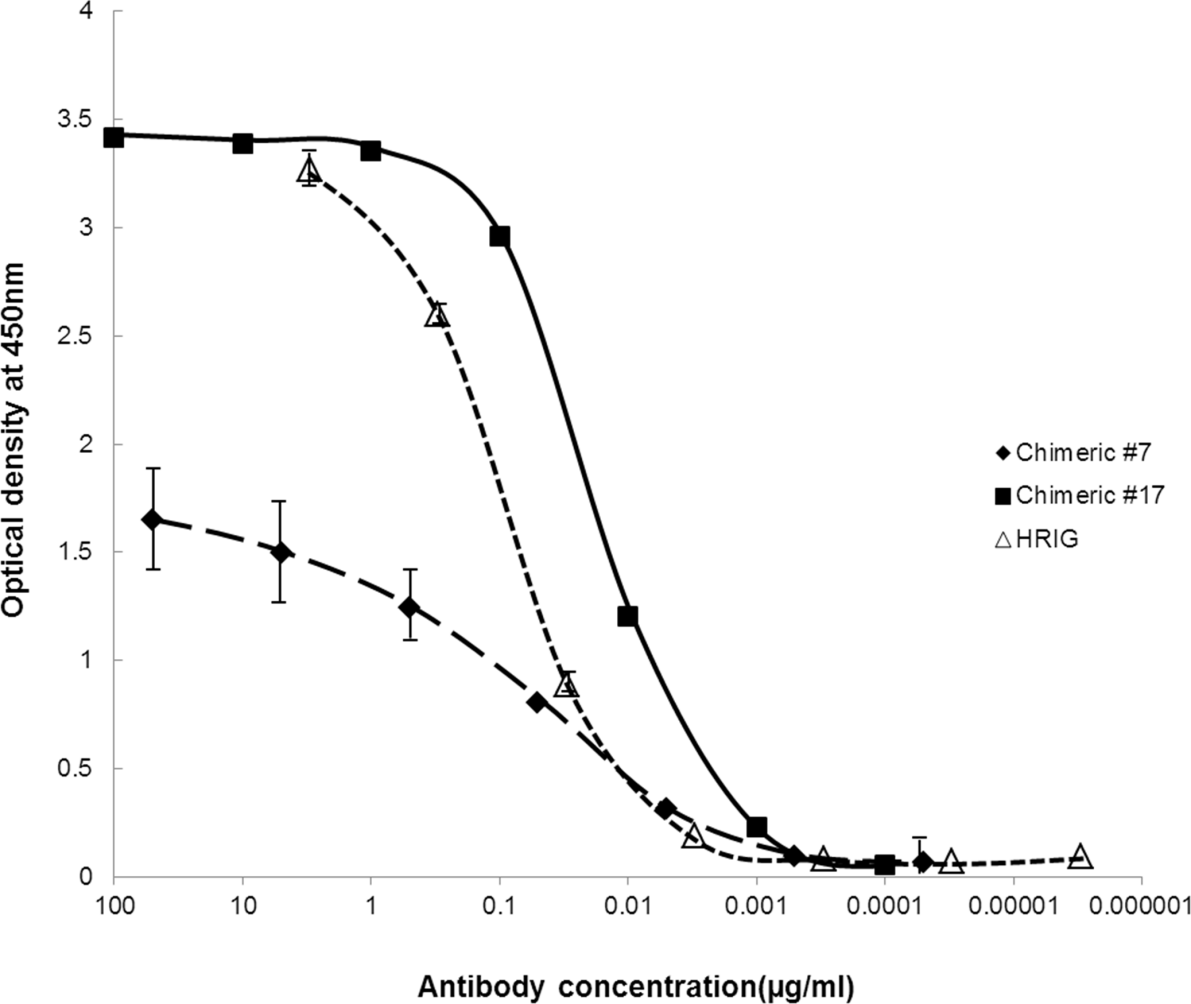
The affinity of chimeric MAbs #7, #17 and HRIG for RABV G protein. Chimeric MAbs #7, #17 and HRIG were incubated in plates coated with 1 μg/mL RABV G protein, and the concentration of each antibody was indicated on the x-axes. Binding to the RABV G protein was expressed as an OD value detected at 450nm. The dots and lines represent the means and standard deviations, respectively, from three independent experiments.

To analyze the binding affinity of chimeric MAbs to RABV G protein, we performed an ELISA assay using purified RABV G protein. Purified G protein (1 μg/mL) was coated on ELISA plates and incubated with chimeric MAbs #7, #17 and HRIG. The EC_50_ values of chimeric MAbs #7 and #17 were determined to be 0.06 μg/mL and 0.02 μg/mL, respectively (Fig 1).

### Generation and characterization of escape viruses from chimeric MAbs #7 and #17

**Fig 2:**
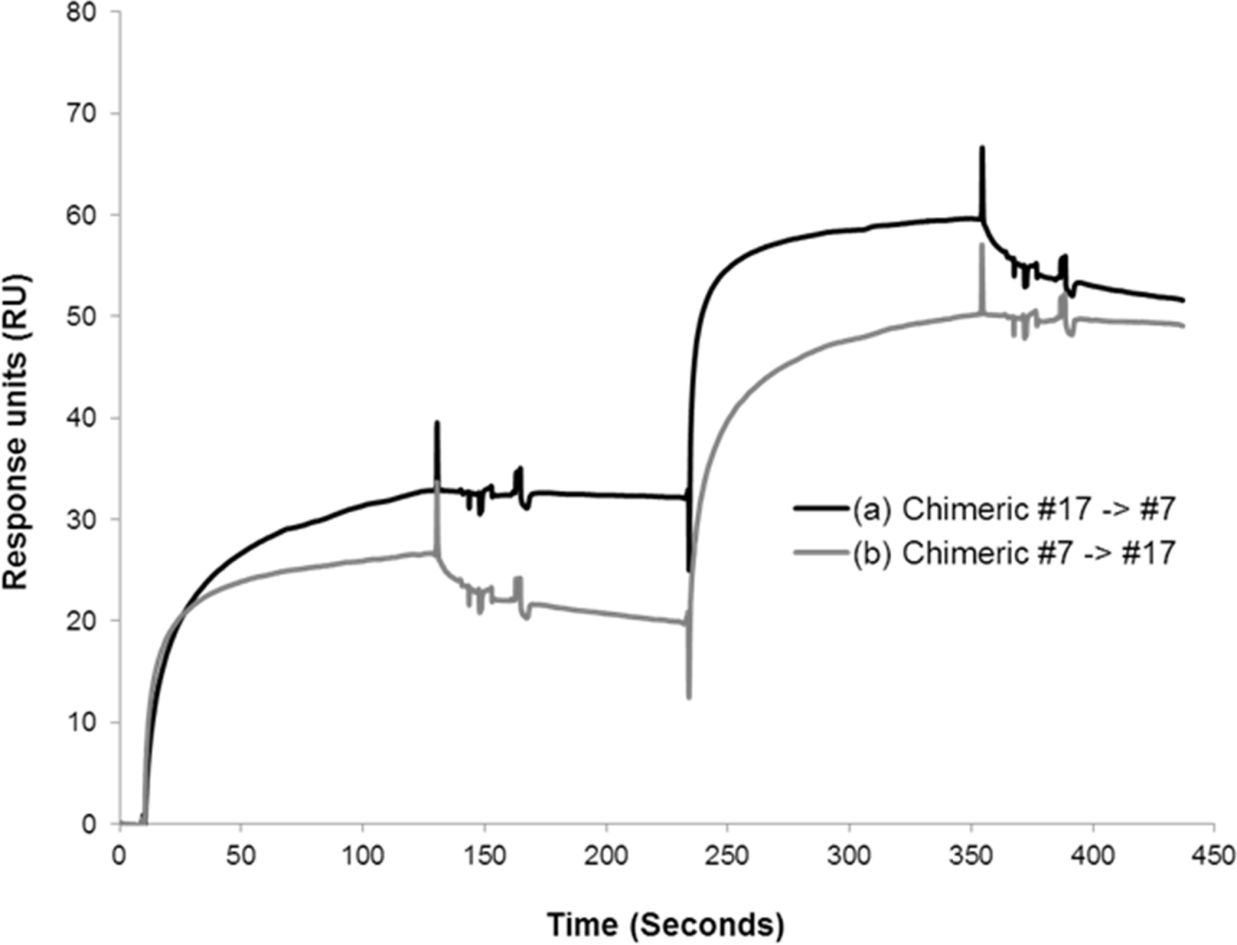
Epitope mapping study by SPR assay. SPR experiments were performed by injecting chimeric MAbs #7 and #17 (1 μM). The time in seconds (x-axis) is plotted against the RU (y-axis). RU levels are indicated for each injected antibody. (a) Injection order: chimeric MAb #17 -> #7. (b) Injection order: chimeric MAb#7 -> #17.

To demonstrate whether chimeric MAbs #7 and #17 bind to different epitopes, an SPR assay was conducted. Epitope mapping studies using Biacore on RABV G protein-immobilized C5 chips showed that chimeric MAbs #7 and #17 bound to different epitopes of the RABV Gprotein (Fig 2). Injection of chimeric MAb #7 resulted in a response of 25 RU. After injection of chimeric MAb #17, an additional increase in the response level (30 RU) was observed, suggesting that binding sites for chimeric MAb #17 were not occupied by chimeric MAb#7. Similar results were observed when the chimeric MAbs were applied in the reverse order.

**Fig 3:**
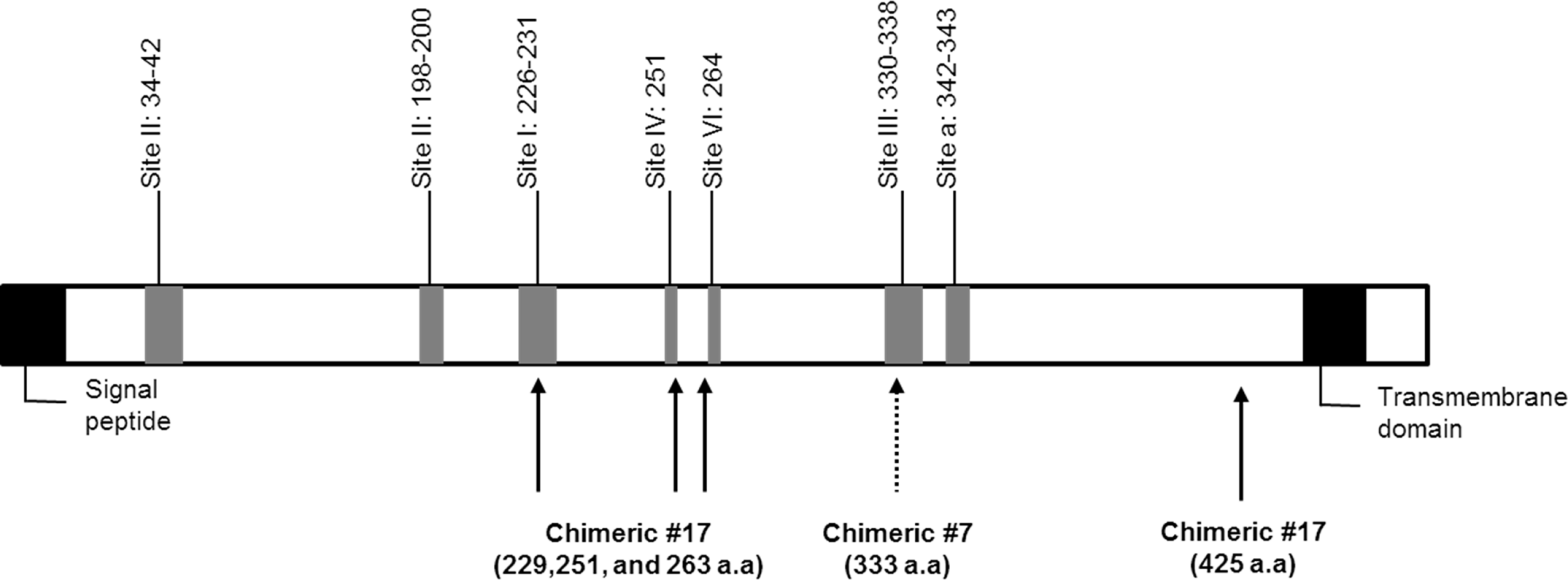
Antigenic site on RABV G protein. A schematic illustration is shown of the antigenic sites of chimeric MAbs #7 and #17. The amino acid numbers are based on the mature G protein minus the signal peptide. The signal peptide and transmembrane domain region are also indicated.

To investigate which antigenic epitopes are recognized by chimeric MAbs #7 and #17, we performed an *in vitro* escape virus generation experiment. Chimeric MAbs #7 and #17-resistant escape viruses were generated as described in Materials and Methods. The G protein-encoding genes of each escape virus were analyzed by direct sequencing of cDNA that was obtained from infected cells. DNA sequence analysis revealed several mutations in the G protein gene. Overall, two escape virus containing different amino acid at same amino acid site for chimeric MAb #7 and four unique escape viruses for chimeric MAb #17 were isolated based on G protein amino acid sequences (Table 3). The R333W and R333L mutations identified in the chimeric MAb #7 escape virus were located in antigenic site III, and the G229E and W251L mutations identified in chimeric MAb #17 escape viruses were located in antigenic sites I and IV, respectively (Fig 3). To confirm whether the viruses resistant to each antibody were neutralized by the other antibody, a cross-neutralization test was conducted. The chimeric MAb #7-resistant viruses were neutralized by chimeric MAb #17; the viruses were also neutralized in the reverse (data not shown). There are several MAbs that recognize antigenic sites I and III [30]. Among them, CR4098 is known to recognize antigenic site III, and site N336, especially, is critical to neutralizing RABV [22]. Because CR4098 has the same antigenic site as chimeric MAb #7, we analyzed the neutralizing activity of chimeric MAb #7 against CR4098-resistant viruses. To isolate CR4098-resistant viruses, CR4098 MAb was produced as described in U.S. patent 8,148,497, and RABV (CVS-11) was grown in the presence of CR4098. We isolated the same N336D mutation identified in previous studies [22]. The neutralizing activity of chimeric MAb #7 against mutant N336D was measured by FAVN assay. CR4098 did not neutralize the N336D mutant, but chimeric MAb #7 neutralized the mutant virus, suggesting that the specific amino acids recognized by each MAb were critical to neutralizing the viruses. Chimeric MAb#7 showed high neutralizing activity (2704 IU/mg) against the CR4098 escape virus.

**Table 3:** Characterization of chimeric MAbs#7 and #17 escape viruses^a^.

Antibody	Virus	Amino acid no^a^	Amino acid change	Condon change
Chimeric MAb #7	#7–1	333	R to W	CGG to TGG
	#7–2	333	R to L	CGG to CTG
Chimeric MAb #17	#17–1	251	W to L	TGG to TTG
		425	K to N	AAA to AAC
	#17–2	229	G to E	GGA to GAA
	#17–3	251	W to L	TGG to TTG
	#17–4	251	W to L	TGG to TTG
		263	F to S	TTT to TCT

^a^ Amino acid numbering is based on the mature protein minus the signal peptide.

### Conservation of RABV epitopes

The residues crucial for binding chimeric mAbs MAbs#7 and #17 were identified by *in vitro* escape experiment. The amino acid variants that were identified as key binding epitopes of chimeric MAb #7 in the escape mutant studies were detected in 68 (6.6%) sequences in a previous report [30]. Although R333K/N/Q containing isolates were detected in several bat lineages from the Americas, there was no R333W or R333L isolates. The key binding site for chimeric MAb #17, antigenic site I (KLCGVL), is highly conserved [21]. In a previous report [21], the glycine at position 229 was conserved in 99% of isolates. Three variants displayed different amino acids at position 229: one isolate had G229R (0.4%) and two isolates were identified as G229E (0.9%).

### *In vivo* protection from lethal RABV infection by chimeric MAbs #7 and #17

**Fig 4:**
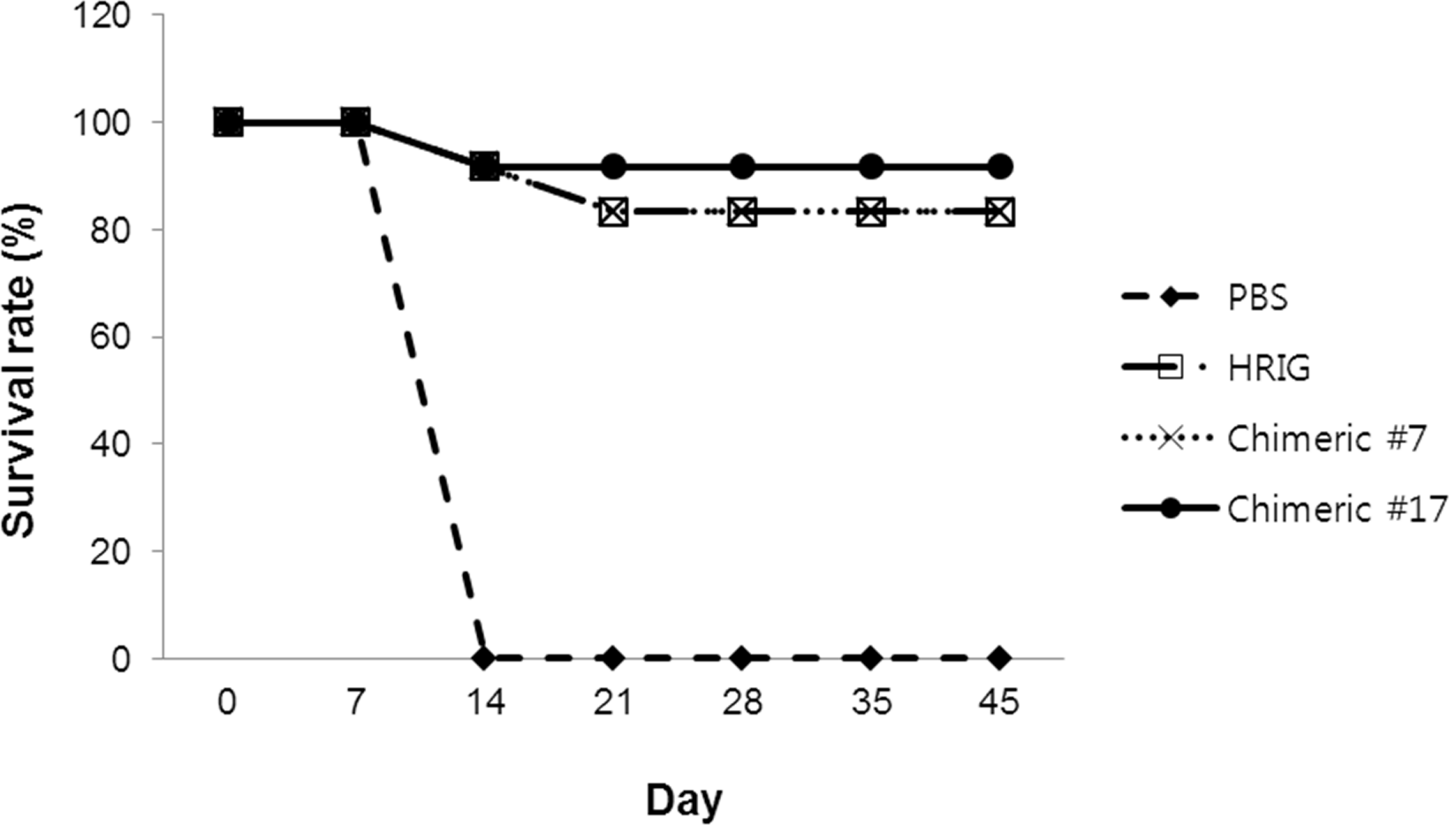
Established MAbs protect hamsters against lethal RABV infection. Syrian hamsters were divided into four groups (n = 12 in each group) and a MICLD_50_ of RABV RV342 from China was injected 1 day before antibody treatment. HRIG and chimeric MAbs #7 and #17 were administered, and animals were monitored daily.

To investigate whether the MAbs could protect against lethal RABV infection *in vivo*, an experiment was performed at U.S. CDC. At 24hrs after administration of a mouse intracerebral lethal dose_50_(MIC-LD_50_) of RABV RV342 from China, HRIG and chimeric MAbs #7 and #17 were administered intramuscularly into the left gastrocnemius muscles. The survival rate in a control group following infection with MICLD_50_ of RV342 decreased at 7 days after injection, and all of the hamsters had died by day 14. However, chimeric MAbs#7 and 17 provided protection against lethal RABV infection, with survival rates of 83% (10/12) and 92% (11/12), and treatment with HRIG resulted in a survival rate of 83% (10/12) (Fig 4). When RABV vaccine was administered together with tested antibodies (chimeras #7 and #17), there was no significant change in the survival rate (data not shown). In conclusion, each of the MAbs tested protected hamsters after exposure to lethal RABV infection.

India and China have the highest burden of human rabies in the world. We analyzed whether chimeric MAbs#7 and #17 could protect against challenge with RABV isolated in India and China. Six virus isolated from different hosts or times in India were tested in *in vivo* mouse protection studies at the National Institute of Mental Health and Neurosciences (NIMHANS) in Bangalore, India. (Table 4). As shown in Table 4(B), 1 month following challenge with these RABVs, all mice treated with HRIG or chimeric MAbs#7 and/or #17 remained healthy, whereas all negative control mice treated with PBS developed signs of rabies varying from 7 to 13 days post infection. Both chimeric MAbs#7 and/or #17 were able to neutralize the RABV isolates from India during *in vivo* challenge in mice. Tested efficacy was the same as commercial HRIG. Additionally, *In vivo* mouse challenge studies were performed by Changchun Veterinary Research Institute using five RABVs isolated from China. Chimeric MAbs #7 effectively protected mice from lethal virus challenges (data not shown).

**Table 4:** Anti- RABV MAbs protect mice against lethal RABV infection.

**(a)**						
**Country**	**Strain**		**Origin**		**Date of isolation**	
India	SV 1		isolated from a dog in Bangalore India		2010.02.02	
	SV 2		isolated from a canine in Bangalore India		2010.02.10	
	SV 3		isolated from a human in Koppal India		2010.9.13	
	SV 4		isolated from canine in Trivandrum India		2011.07.18	
	SV 5		isolated from canine in Trivandrum India		2011.07.18	
	SV 6		isolated from canine in Trivandrum India		2011.10.14	
**(b)**						
**Challenge rabies virus**	**Survival rate (%)**					
	**Chimeric MAb #7**		**Chimeric MAb #17**		**HRIG**	**PBS**
	**20IU/kg** **(0.014mg/kg)**	**40IU/kg** **(0.028mg/kg)**	**20IU/kg** **(0.13mg/kg)**	**40IU/kg** **(0.26mg/kg)**	**20IU/kg**	
CVS-11	100%	100%	100%	100%	100%	0%
SV1	100%	100%	100%	100%	100%	10%
SV2	100%	100%	100%	100%	100%	10%
SV3	100%	100%	100%	100%	100%	0%
SV4	100%	100%	100%	100%	100%	20%
SV5	100%	100%	100%	100%	100%	0%
SV6	100%	100%	100%	100%	100%	10%

Mice were challenged with RABV isolated in India (a) at 24 hrs before antibody treatment. Animals were injected with several concentrations of antibodies, shown in (b). Control groups received either phosphate-buffered saline or human rabies immune globulins. Animals were monitored daily for 1 month. The survival rates are shown in a table (b).

### Immunogenicity of chimeric MAbs #7 and #17

Immune response against biologic affects clinical efficacy and safety. It is important to analyze the immunogenicity of biologics. Therefore, we determined the immunogenicity of chimeric MAbs #7 and #17 by measuring their capacities to induce CD4+T cell responses. CD4+ T cell responses after inoculation of chimeras #7 and #17 were measured using peripheral blood mononuclear cells (PBMCs) from a cohort of 50 healthy donors. These donors were selected to best represent the number and frequency of HLA-DR allotypes expressed in the world population. Analysis of the allotypes expressed in the cohort against those expressed in the world population revealed that coverage of >80% was achieved, and that all major HLA-DR allotypes were well represented (S1 Fig).

**Fig 5:**
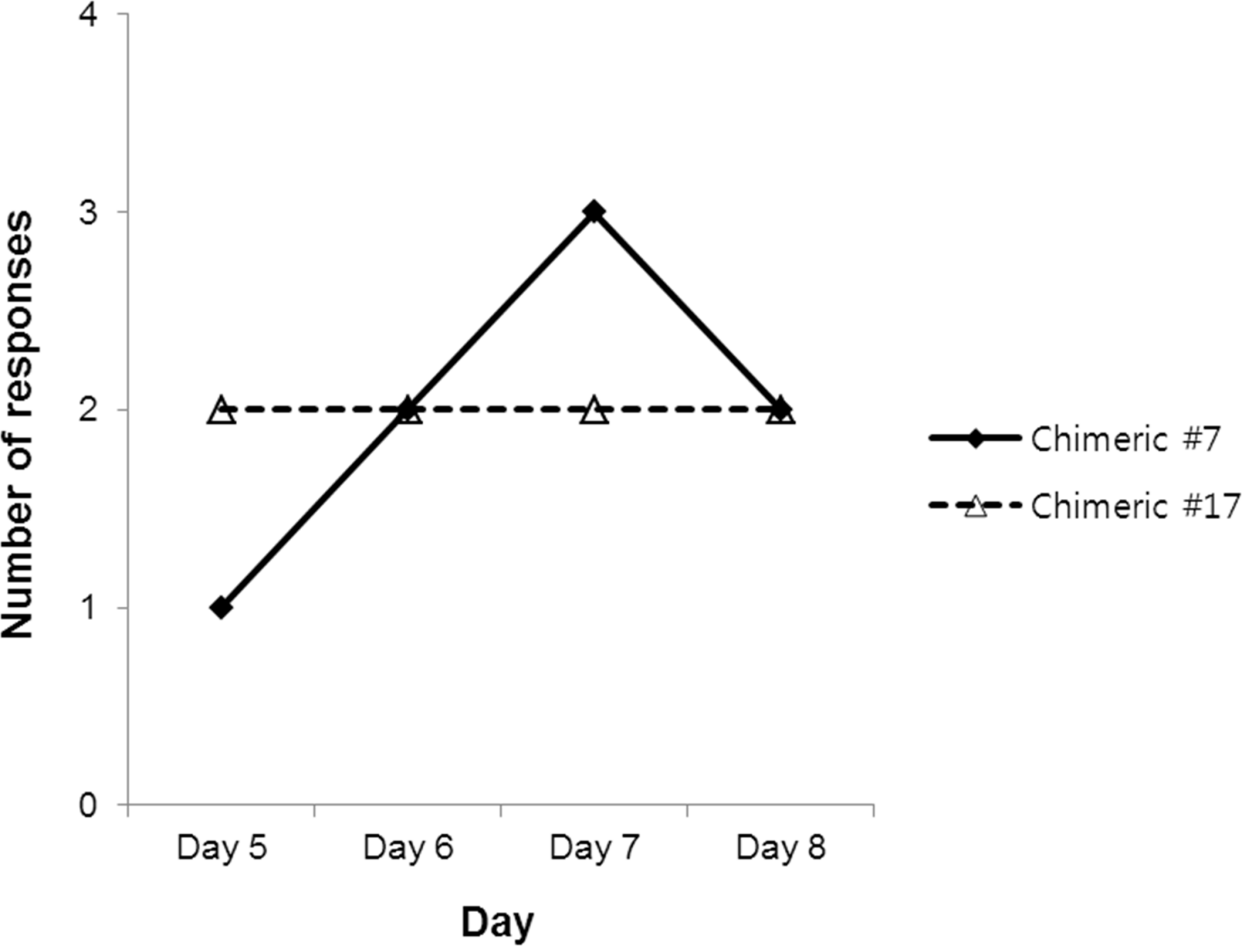
The number of T cell proliferative responses detected at four time points. T cell proliferation was assessed by [^3^H]-thymine uptake on days 5, 6, 7, and 8 after incubation with the antibodies, and the number of positive responders was plotted.

Culture medium or chimeric MAbs #7 and #17 were added to the PBMCs and then were incubated for 8 days at 37°C with 5% CO_2_. T cell activation was detected by proliferation ([^3^H]-thymine uptake) and IL-2 cytokine secretion (ELISpot) assays. Keyhole Limpet Haemocyanin (KLH) and Phytohaemagglutanin (PHA) were used as a positive control in T cell activation and ELISpot assays. The value of responses in test wells with antibodies relative to those measured in non-treated wells was the outcome criterion. The value is presented as a stimulation index (SI), with an SI equal to or greater than 2 (SI ≥ 2.00, and, where included, borderline SI ≥ 1.90) was considered positive. Chimeric MAbs #7 and #17 induced a T cell proliferation response in five and four donors, respectively, among 50 donors, including one borderline response to each antibody, and the mean SIs were positive (SI ≥ 2.00). T cell responsive donors showed that both antibodies induced a low mean SI (Table 5). The number of positive T cell responses that were observed on each day of the four time courses was summarized Fig 5 The results showed that the number of T cell responses on day 5 was low, suggesting that the number of epitope-specific precursor T cells present in healthy donors was relatively low. For chimeric MAb #7, a positive IL-2 ELISpot response was detected in five donors, four of which showed T cell proliferation. Chimeric MAb #17 showed a positive IL-2 ELISpot response against PBMCs from three of the four donors that showed T cell proliferation. Similar to the proliferation assay data, the mean SI of positive T cell responses in the IL-2 ELISpot assay was low, ranging between 1.98 and 2.24 (Table 5). Analysis of the combined datasets from these two assays indicated that the overall frequencies of donors responding in both the proliferation and IL-2 ELISpot assays for chimeric antibodies #7 and #17 were 8% and 6%, respectively.

**Table 5:** Summary of T cell activation assay.

Sample	Proliferation assay		ELISpot assay		Both assays
	% of positive responders	Mean SI value	% of positive responders	Mean SI value	% of positive responders
Chimeric MAb #7	10	2.27	8	2.24	8
Chimeric MAb #17	8	2.5	6	1.98	6
Positive control	80	6.11	88	3.19	76

SI: stimulation index

SI index greater than or equal to 2 (SI ≥ 2.00), where SI = the mean of test wells (cpm or spw)/baseline (cpm or spw).

## Discussion

At present, HRIG and ERIG are used for PEP of patients exposed to RABV. Since these immune globulins were harvested from sera obtained from immunized humans or horses, their quantities and availability are limited, especially in many developing countries. In addition, blood-derived products raise safety concerns such as the presence of unknown pathogens. HRIG, specifically, is available in limited quantities. Hence, HRIG is very expensive. Some adverse effects have been reported in ERIG-treated people [39]. Thus, MAbs are an attractive alternative to RIG.

To develop alternatives for RIG, we screened hybridoma products to identify potent MAbs that neutralized RABV of multiple variants. The neutralizing potencies from the MAbs of candidate hybridomas were determined against lyssaviruses of various genotypes and phylogroups. Previously identified MAbs were selected by testing them against 20 RABVs [23]. For comparison, we tested the neutralizing activity of the MAbs produced by hybridoma against 50 RABVs isolated from different hosts and countries. We selected two hybridomas, #2-21-23 and #62-71-3, whose MAbs neutralized 86% and 92% of tested viruses (S1 Table). The IgG variable regions from selected hybridomas were amplified and cloned into vectors containing a human antibody constant region. CHO-K1 cells were used to produce chimeric MAbs. These stable cell lines consistently expressed high quantities of MAbs. Although the amount was sufficient to adapt towards large-scale production (~g/L), in the future, process optimization will be tried to improve expression further to obtain lower-cost products to compete with RIG. We only produced chimeric MAbs #7 and #17 at 3L scale bioreactor. Therefore, we need to optimize the process at large scale production (e.g. 15,000L).

The binding affinity of each MAb to RABV G protein was determined by ELISA (Fig 1). To conduct the ELISA, we developed and optimized the process for purifying RABV G protein. The purification process enhanced significantly the G protein yield compared to that from a previously reported protocol [40] (data not shown). The concentrations of MAbs that showed half-maximal binding were 60 nM and 20 nM. The values were smaller than those of the standard antibody (HRIG), which had a 90 nM EC_50_ value (Fig 1).

The important criterion to select MAbs to replace RIG is the binding specificity. Thus, two different MAbs directed at different antigenic sites are needed [41]. The epitope sites of the two MAbs were clearly different, as demonstrated by the Biacore assay (Fig 2). Among the antigenic sites, the key residues required to neutralize virus were identified by *in vitro* escape experiments. Chimeric MAb #7 targeted antigenic site III, and R333W/L mutants were not neutralized by chimeric MAb #7 (Table 3). Some human MAbs targeted antigenic site III, including CR4098, RAB1, and 17C7 [23,33,34]. To investigate whether chimeric MAb #7 could neutralize mutant viruses generated by other MAbs targeting the same antigenic site, we constructed the CR4098 MAb and performed an *in vitro* escape experiment. We isolated the same mutant virus against CR4098, N336D. This indicated that the constructed CR4098 MAb was identical to the published MAb. Although CR4098 and chimeric MAb #7 targeted the same antigenic site, chimeric MAb #7 neutralized the CR4098 mutant virus, N336D. This result suggested that one or a few amino acids in the antigenic site determined the neutralizing activity of the MAb. The variants that had a different amino acid at the residue crucial for binding chimeric MAb #7, R333, were detected in 68 sequences in a previous report [31]. Although R333K/N/Q isolates were detected in several bat RABV lineages from the Americas, there was no R333W or R333L isolate found. Whether R333K/N/Q variants can be neutralized by chimeric MAb #7 remains to be tested. In contrast to chimeric MAb #7, chimeric MAb #17 seems to target an overlap of two antigenic sites I and IV (Table 3). Five mutant viruses that escaped chimeric MAb #17 were isolated in an *in vitro* escape experiment. The two mutants showed a G229E substitution in antigenic site I, and three mutants showed a W251L change in antigenic site IV that has been found in unrelated mutants, K425N and F263S. Almost all reported MAbs bind to one antigenic site [31]. However, there was a report that some MAbs bound overlapping epitopes [42]. Antigenic site mapping of candidate MAbs by cross-competition using reference MAbs with known epitope specificity was performed in the previous study. Some candidate MAbs only competed against particular antibodies, even though they had the same antigenic sites. Taken together, this result and that of our *in vitro* escape experiment show that antigenic sites I and IV of the RABV G protein are critical to binding chimeric MAb #17. Chimeric MAb #17 was derived from hybridoma #62-71-3 supplied by the U.S. CDC, which was previously described [11, 32]. Both et al. established a plant-based production system for a chimeric version of MAb#62-71-3. Although the origin of the hybridoma used to construct chimeric antibodies was the same, the chimeric MAbs showed different characteristics. Both K226 and G229 amino acids were critical to neutralizing virus. However, we did not identify K226 mutant virus in the *in vitro* escape experiment with chimeric MAb #17. Further study is needed to understand the difference between these chimeric MAbs.

The efficacy of chimeric MAbs was confirmed via *in vivo* hamster and mouse challenge studies (Fig 4 and Table 4). Chimeric MAb #17 showed higher efficacy than HRIG, even though chimeric MAb #17(12I U/kg) was administered at a lower IU/kg than HRIG (20 IU/kg). This result correlated with the result obtained with chimeric MAb #17, which showed higher neutralizing activity than HRIG against the challenge virus, RV342. Chimeric MAbs #7 and #17 showed lower neutralizing antibody titers than HRIG against only three and four viruses, respectively. However, some viruses that were neutralized weakly by chimeric MAb #7 were strongly neutralized by chimeric MAb #17. Thus, we suggest that a cocktail of chimeric MAbs #7 and #17 should protect against all tested viruses, except for a virus isolated from a skunk in the USA, which was not neutralized by either MAb. In the hamster challenge study, an excessive amount of chimeric MAb #7 was used, and we did not test *in vivo* protection using a lower amount. However, the mouse challenge study using viruses isolated in India or China showed the protective potential of chimeric MAb #7 (Table 4). Similar to HRIG, 20 IU/kg of chimeric MAb #7 protected against lethal challenge by several viruses. To confirm the potential to use chimeric MAbs #7 and #17 in a cocktail, an interference test of the individual antibodies together is needed, and *in vivo* protection studies using a challenge virus that is weakly neutralizing by one MAb but strongly neutralized by the other MAb, i.e., bat, California, USA and skunk, Texas, USA, are needed. If there is no interference and they protect against the virus mentioned above, a cocktail of chimeric MAbs #7 and #17 offers an attractive alternative to standard MAbs currently in use.

If immunogenic *in vivo*, the MAb might elicit a weak clinical effect, even if they showed potent neutralization *in vitro*. To reduce the immunogenicity of MAbs, other groups have developed human MAbs [21, 22, 23, 24, 42]. We also isolated human MAbs that showed broad-spectrum neutralization of RABV by phage display and B-cell sorting. The isolated human MAbs demonstrated higher neutralizing activity than previously reported MAbs. Even though chimeric MAbs #7 and #17 are hybrids of mouse and human antibodies, the immunogenicity of these indicates that the overall potential risk of adverse events is low. In general, biologics inducing a > 10% T cell proliferation response were associated with a significant risk of adverse immunogenicity in the clinic.

In conclusion, we have identified two potent monoclonal antibodies, chimeric MAbs #7 and #17. The antibodies recognize non-overlapping, non-competing epitopes, cross-protect against each other’s escape viruses, and neutralize a broad range of field isolates of RABV. In addition, *in vivo* efficacy studies using either of the MAbs resulted in protection equivalent to that of HRIG when animalswere challenged with a lethal RABV dose. Taken together, these results indicate that the selected MAbs could replace RIG as an alternate in future human PEP.

## Supporting information

S1 Fig Comparison of the frequency of donor allotypes expressed in our study and the world population. (PDF)

S1 Table Breadth of hybridoma supernatant neutralization of RABV field isolates. (PDF)
