# Supplementary material for: Development and characterization of novel chimeric monoclonal antibodies for broad spectrum neutralization of rabies virus
Source: PLoS One. 2017 Oct 18;12(10):e0186380. doi: 10.1371/journal.pone.0186380 (PMC5646816; doi:10.1371/journal.pone.0186380)

S1 Figure. Comparison of the frequency of donor allotypes expressed in our study and the world population

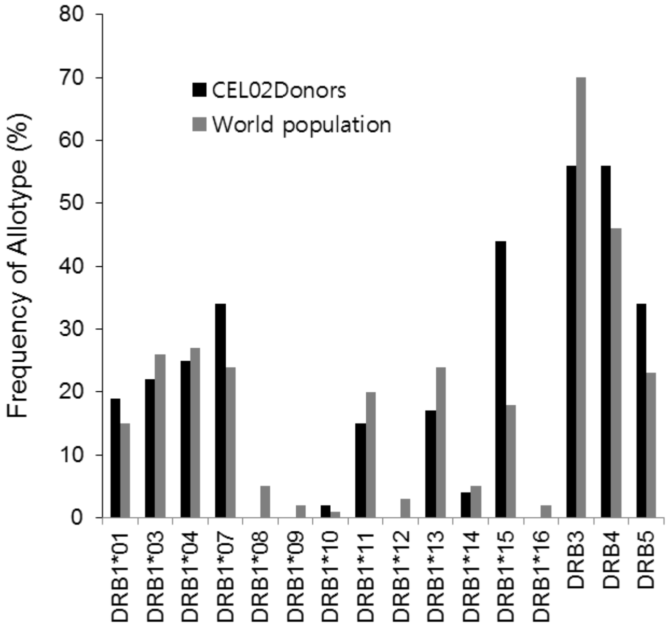

Supplement: S1 Fig — (PDF) [file pone.0186380.s001.pdf]
