# Supplementary material for: Development and characterization of novel chimeric monoclonal antibodies for broad spectrum neutralization of rabies virus
Source: PLoS One. 2017 Oct 18;12(10):e0186380. doi: 10.1371/journal.pone.0186380 (PMC5646816; doi:10.1371/journal.pone.0186380)
Supplement: S1 Table — (PDF) [file pone.0186380.s002.pdf]

S1 table. Breadth of hybridoma supernatant neutralization of RABV field isolates

No.	Lyssavirus (Isolated source, country)	2-21-8	2-21-23	2-21-14	62-71-3	62-80-6
1	Rabid Cow	+[a]	+	+/-[b]	+	+
2	Mongoose, South Africa	+	+	+	+	+
3	Skunk, California, USA	+/-	N.D[d]	+	N.D	+
4	Dog, Tunissia	+	+	+	+	+
5	Dog, Gabon,Africa	+	+	+	+	+/-
6	Gray fox, Texas,USA	+	+	+	+	+
7	Dog, Thailand	+	+	+	+	+
8	Dog, Mexico	+	+	+	+	+
9	Human/dog, Philippines	+	+	+	+	+/-
10	Bat, Mexico	+	+	+	N.D	+/-
11	Bat, Brazil	+	+	+	N.D	+
12	Dog, Philippines	+	+	+	+	+
13	Bat, Washington, USA	+	+	+	+	+
14	Bat, California, USA	+/-	-[c]	+/-	+	+
15	Dog, Argentina	+	+	+	+	+
16	Skunk, Texas, USA	+	+	+	-	-
17	Raccoon, Southeast, USA	+	+	+	N.D	+/-
18	Dog, China	+	+	+	+	+
19	Cow/dog, China	+	+	+	+	+/-
20	Coyote, Texas, USA	+	+	+	+	+
21	Human/dog, United Kingdom	+	+	N.D	N.D	N.D
22	Bat, California, USA	+	+	N.D	+	N.D
23	Bat, New York, USA	+	+	N.D	N.D	N.D
24	Bat, Pennsylvania, USA	+	+	N.D	+	N.D
25	Bat, Alabama, USA	+	+	N.D	+	N.D
26	Bat, Arizona, USA	-	-	N.D	+	N.D
27	Bat, Virginia, USA	-	N.D	N.D	+	N.D
28	Bat, Tennessee, USA	+	N.D	N.D	+	N.D
29	Bat, Tennessee, USA	-	N.D	N.D	+	N.D
30	Skunk, Texas, USA	+/-	+	N.D	N.D	-

31	Arctic Fox, Alaska, USA	N.D	+	N.D	-	+/-
32	Raccoon dog, Russia, Far East	+	+	N.D	+	+
33	Dog, India	+	+	+	+	+/-
34	Mongoose, Puerto-Rico	+	+	N.D	+	+
35	Gray Fox, Arizona, USA	+/-	+	N.D	+	N.D
36	Skunk, Wisconsin, USA	+	+	N.D	+	N.D
37	Dog /Coyote, Texas, USA	N.D	+	N.D	+	N.D
38	Human/wolf, Russia, Arctic	N.D	+/-	N.D	N.D	+/-
39	Bat, Tennessee, USA	N.D	+	N.D	+	+
40	Dog, India	+	+	+	N.D	-
41	Bat, Tennessee, USA	+/-	+/-	-	+	-
42	Cow, Sri Lanka	+/-	+/-	N.D	+	-
43	Bat, Washington, USA	+/-	+/-	N.D	+	N.D
44	Bat, Australia	+	+	N.D	-	N.D
45	Bat, Australia	+	+	N.D	+	N.D

Neutralizing potency against each RABV field isolates was measured in a standard RFFIT and indicated as follows

[a]: +, Neutralizing potency of MAbs > 20% of HRIG's neutralizing potency

[b]: +/−, Neutralizing potency of MAbs < 20% of HRIG's neutralizing potency

[c]: -, No neutralization, [d]: Not determined
